# Failure of Amino Acid Homeostasis Causes Cell Death following Proteasome Inhibition

**DOI:** 10.1016/j.molcel.2012.08.003

**Published:** 2012-10-26

**Authors:** Amila Suraweera, Christian Münch, Ariane Hanssum, Anne Bertolotti

**Affiliations:** 1 MRC Laboratory of Molecular Biology, Hills Road, Cambridge CB2 0QH, UK; 2 Department of Cell Biology, Harvard Medical School, Boston, MA 02115, USA

## Abstract

The ubiquitin-proteasome system targets many cellular proteins for degradation and thereby controls most cellular processes. Although it is well established that proteasome inhibition is lethal, the underlying mechanism is unknown. Here, we show that proteasome inhibition results in a lethal amino acid shortage. In yeast, mammalian cells, and flies, the deleterious consequences of proteasome inhibition are rescued by amino acid supplementation. In all three systems, this rescuing effect occurs without noticeable changes in the levels of proteasome substrates. In mammalian cells, the amino acid scarcity resulting from proteasome inhibition is the signal that causes induction of both the integrated stress response and autophagy, in an unsuccessful attempt to replenish the pool of intracellular amino acids. These results reveal that cells can tolerate protein waste, but not the amino acid scarcity resulting from proteasome inhibition.

## Graphical abstract

**Graphical Abstract:**

**Figure:**
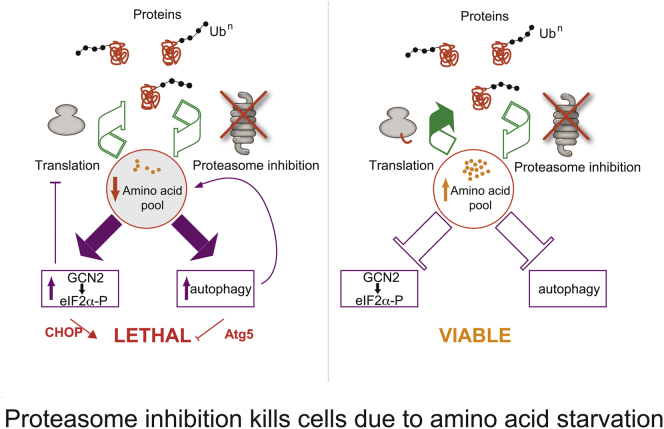

**Highlights:**

► Proteasome inhibition results in a lethal amino acid scarcity ► Amino acid supplementation rescues death caused by proteasome inhibition ► Proteasome-inhibited cells die by failure to maintain amino acid homeostasis ► Proteasome-inhibited cells can survive with their protein waste

## Introduction

The controlled degradation of proteins by the proteasome is essential in all cells and impairment of the ubiquitin-proteasome system contributes to many pathological conditions (Hershko and Ciechanover, 1998; Finley, 2009). Although the proteasome inhibitor Bortezomib is used for the treatment of multiple myeloma (Hideshima et al., 2001; Goldberg, 2007; Navon and Ciechanover, 2009), it remains unclear how proteasome failure kills cells.

Because the ubiquitin-proteasome system controls the degradation of a large number of cellular proteins including short-lived, regulatory, and damaged or misfolded proteins (Hershko and Ciechanover, 1998; Schwartz and Ciechanover, 2009), it has been assumed that accumulation of no-longer needed proteins underlies the toxicity of proteasome inhibition. However, how such proteins become harmful to cells and organisms remains unclear. Failure to degrade key regulatory proteins could also perturb cellular function. The transcription regulator NF-κB is one such regulatory protein activated by proteasome cleavage (Palombella et al., 1994). NF-κB is constitutively activated in multiple myeloma and thus was thought to be a prominent target of proteasome inhibitors (Schwartz and Ciechanover, 2009). However, as selective inhibition of NF-κB does not recapitulate the cytotoxic effects of proteasome inhibition (Hideshima et al., 2002), alternative mechanisms need to be considered.

The function of the proteasome in protein waste disposal has been extensively studied (Hershko and Ciechanover, 1998; Finley, 2009). In addition to this well-described function, the proteasome also recycles amino acids. The importance of this aspect of proteasomal degradation for normal cell and organismal function is unclear. In contrast, recycling amino acids is a well-established function of autophagy in starving cells (Nakatogawa et al., 2009). Under conditions of amino acid starvation, both autophagy and proteasomal degradation are required to maintain adequate amino acid levels for sustaining protein synthesis (Onodera and Ohsumi, 2005; Vabulas and Hartl, 2005). Despite the established importance of protein recycling under conditions of severe nutrient deficiency, it is unclear whether protein degradation contributes an important fraction of the supply of amino acids under physiological conditions.

Because the mechanisms underlying the toxicity resulting from proteasome inhibition are unclear, and as it is unknown whether protein degradation contributes an essential fraction of the intracellular amino acid pool under normal conditions, we examined whether the lethality resulting from proteasome inhibition is mediated by perturbation of amino acid homeostasis. In three model systems—yeast, mammalian cells, and *Drosophila—*we find that proteasome inhibition causes a lethal shortage of amino acids and the lethality resulting from proteasome inhibition is rescued upon amino acid supplementation, without any detectable decrease in the levels of proteasome substrates in proteasome-inhibited cells.

## Results

### Failure of Amino Acid Homeostasis Causes Cell Death following Proteasome Inhibition in Yeast

**Figure 1:**
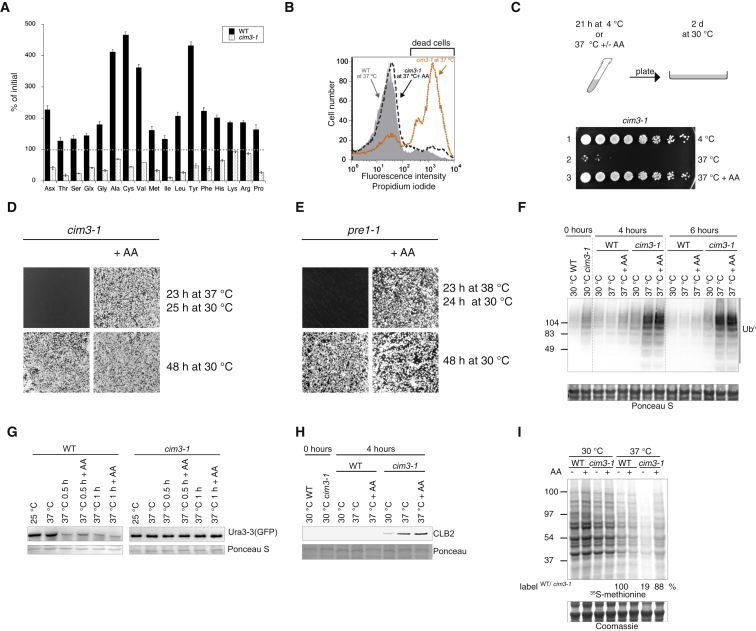
Amino Acid Insufficiency Mediates the Lethality Resulting from Proteasome Inhibition in Yeast Cells (A) Changes in the content of cellular free amino acids in *cim3-1* or wild-type (WT) cells after 5 hr at 37°C in rich medium (YPD). Data are means ± SD (n = 3), normalized to cell number. Significance of all values in wild-type versus mutant cells: p ≤ 0.001. Cells were in exponential growth at 30°C before the temperature switch. Asx: Asn, Asp. Glx: Glu, Gln. (B) Cell death monitored by flow cytometry analysis of propidium iodide labeled yeast cells after 21 hr at 37°C, in the presence of 40 g/l casamino acids (AA) where indicated. (C) Viability of *cim3-1* cells exposed to 37°C for 21 hr in liquid cultures (YPD), in the presence of 40 g/l casamino acids (AA) where indicated or kept at 4°C, assessed by spotting serial dilutions on rich media and incubation at 30°C for 2 days. (D and E) Viability of *cim3-1* and *pre1-1* cells on YPD plates exposed to the indicated temperatures for the indicated times, with or without casamino acids (AA, 40 g/l). A total of 2,000 cells were plated in each condition. (F) Polyubiquitin immunoblots (Ub^n^) from lysates of wild-type or *cim3-1* cells, cultured as indicated. Ponceau staining of the membrane. (G) Immunoblots of GFP-tagged Ura3-3 from lysates of the indicated cells treated with cycloheximide as in Lewis and Pelham (2009) and cultured as indicated. (H) Accumulation of Cyclin B2 (CLB2) in *cim3-1* cells at 37°C. CLB2 immunoblots from lysates of wild-type or *cim3-1* cells, grown in rich medium with or without casamino acids (AA, 40 g/l) for the indicated times at 30°C or 37°C. Ponceau staining of the membrane prior to probing with antibodies. (I) Autoradiogram of [^35^S]methionine-labeled proteins in lysates resolved by NuPage after a 8 min pulse labeling of exponentially growing cells cultured as indicated and quantification of labeled proteins. Lower panel is a photomicrograph of the Coomassie-stained gel. Representative results of at least three independent experiments are shown. See also Figure S1.

We first assessed whether amino acid homeostasis was altered following proteasome impairment in yeast. As expected (Ghislain et al., 1993), exposure to 37°C for 20 hr was lethal to the yeast proteasome mutant *cim3-1*, harboring a thermosensitive mutation in the regulatory subunit Rpt6 (Figure S1A available online). We next monitored the levels of free amino acids in yeast cells following proteasome inhibition. Exponentially growing yeast cells were inoculated at an optical density of 0.2 and grown at 30°C in the standard rich medium yeast peptone dextrose (YPD) to insure that the yeast cells were replete with nutrients. Five hours after the switch to 37°C, the levels of most amino acids increased in wild-type cells (Figure 1A). This is likely the result of the heat shock, which is known to attenuate protein synthesis (Richter et al., 2010). Surprisingly, however, the levels of most free amino acids decreased in *cim3-1* cells after 5 hr at 37°C (Figure 1A). Unlike wild-type cells, *cim3-1* cells did not grow at 37°C (Figure S1B), indicating that the decrease in intracellular amino acids in *cim3-1* cells was not due to an exhaustion of the nutrient supply from the media as a result of cell growth. Thus, inhibition of proteasomal degradation, under conditions of nutrient sufficiency, decreases the levels of intracellular amino acid in yeast cells.

We then investigated whether increasing the supply of amino acids could rescue *cim3-1* cells. Exponentially growing wild-type or *cim3-1* yeast cells were inoculated at an optical density of 0.2 and cultured in the standard rich medium YPD for 21 hr, labeled with propidium iodide to reveal dead cells, and analyzed by flow cytometry. Unlike wild-type cells, the vast majority of *cim3-1* cells died after 21 hr of culture at 37°C in rich media (Figure 1B). supplementation with amino acids prevented the death of the *cim3-1* cells exposed to 37°C (Figure 1B). To determine whether such cells were viable, they were spotted on rich plates and grown at the permissive temperature. Unlike the nonsupplemented *cim3-1* cells, *cim3-1* cells that had been exposed to 37°C in the presence of additional amino acids were viable and gave rise to the same number of colonies as wild-type cells at 30°C (Figure 1C). This revealed that amino acid supplementation prevents death of proteasome-inhibited yeast cells. This rescuing effect occurs without promoting cell growth because the number of viable cells exposed to 37°C in the presence of amino acids equaled the number of cells prior exposure to 37°C (Figure 1C, compare lane 1 to lane 3). Supplementation with amino acids also protected *cim3-1* cells exposed to 37°C on plates (Figure 1D), as well as the proteasome mutant *pre1-1* cells, harboring a thermosensitive mutation in the β4 subunit of the 20S proteasome (Heinemeyer et al., 1991) (Figure 1E), but not the *ndc80-1* mutant cells (Figure S1C), which harbors a thermosensitive mutation unrelated to the proteasome, in a spindle body component (Wigge et al., 1998).

Although rescuing viability, addition of amino acids did not noticeably decrease the levels of polyubiquitinated proteins (Figure 1F) or the unstable GFP-tagged Ura3-3 (Lewis and Pelham, 2009) that accumulated in *cim3-1* cells at 37°C (Figure 1G). Thus, amino acid supplementation rescues from the lethality resulting from proteasome inhibition without promoting a recovery of proteasome function.

Proteasomal degradation is required for progression through the cell cycle (Hershko, 2005) and at nonpermissive temperature, *cim3-1* cells stop dividing (Ghislain et al., 1993) and (Figure S1B) and accumulate cyclins, such as CLB2 (Ghislain et al., 1993). Although rescuing the viability of proteasome-inhibited cells, amino acids supplementation did not alleviate the growth arrest resulting from proteasome inhibition (Figure S1B). Consistently, the levels of CLB2 were indistinguishable in *cim3-1* cells at 37°C with or without amino acids supplementation (Figure 1H). This confirmed that amino acids did not rescue proteasomal degradation or the resulting growth impairment in proteasome inhibited cells, while rescuing viability.

We next examined if the marked decrease in amino acid noted in *cim3-1* cells impacted on protein synthesis. Note that, as expected (Richter et al., 2010), heat shock reduced protein synthesis in wild-type cells (Figure 1I). Strikingly, we found that in *cim3-1* cells, protein synthesis at 37°C was reduced to 19% of the wild-type cells exposed to the same temperature (Figure 1I). Supplementation with amino acids rescued translation rates in the *cim3-1* cells to nearly wild-type levels (Figure 1I).

### Amino Acid Supplementation Rescues the Deleterious Consequences of Proteasome Inhibition in Mammalian Cells

**Figure 2:**
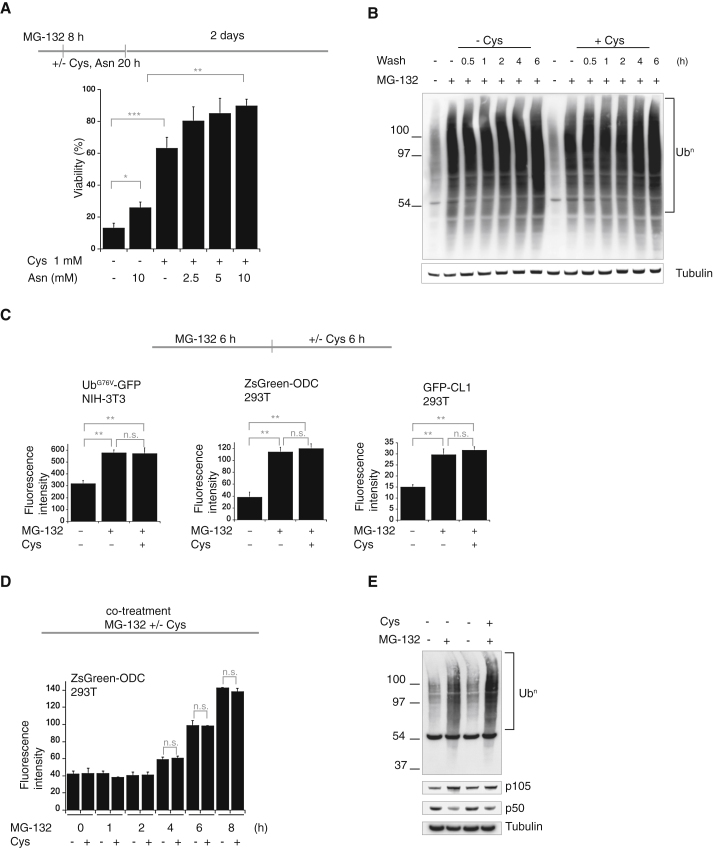
Failure of Amino Acid Homeostasis Causes Cell Death following Proteasome Inhibition (A) Viability of NIH 3T3 cells, assessed by the reduction of WST-8 into formazan, after 2 days of growth, following treatment with 10 μM MG-132 for 8 hr and a 20 hr washout in regular medium, with or without cysteine (Cys) and/or asparagine (Asn). Data are means ± SD (n = 4). ^∗^p ≤ 0.01, ^∗∗^p ≤ 0.001, and ^∗∗∗^p ≤ 0.0001. n.s., not significant. (B) Polyubiquitin (Ub^n^) and tubulin immunoblots of lysates from NIH 3T3 cells exposed to 10 μM MG-132 treatment for 6 hr, followed by a washout in regular medium, with or without 1 mM Cys supplementation, for the indicated time. (C) Fluorescence of proteasome reporter-substrates Ub^G76V^-GFP (Dantuma et al., 2000), ZsGreen-ODC (Hoyt et al., 2005), and GFP-CL1 (Gilon et al., 1998; Bence et al., 2001) in NIH 3T3 or 293T cells, following treatment with 10 μM MG-132 and a washout for the indicated time, with or without Cys. Data are means ± SD (n = 3). ^∗^p ≤ 0.01, ^∗∗^p ≤ 0.001, and ^∗∗∗^p ≤ 0.0001. n.s., not significant. (D) Fluorescence of proteasome reporter-substrates ZsGreen-ODC (Hoyt et al., 2005) following treatment with 10 μM MG-132 either alone or together with 1 mM Cys where indicated. (E) Polyubiquitin (Ub^n^), tubulin, and NF-κB (p105 and p50) immunoblots of lysates of 293T cells overexpressing p105 and treated with 10 μM MG-132 for 8 hr, either alone or together with 1 mM Cys, where indicated. Representative results of at least three independent experiments are shown. See also Figure S2.

Because proteasome function is conserved in evolution, we next examined whether inhibition of the proteasome altered amino acid homeostasis in mammalian cells. Treatment of mammalian NIH 3T3 cells with the proteasome inhibitors MG-132 or Bortezomib decreased the levels of the amino acids asparagine/aspartate and cysteine, whereas the levels of other amino acids remained largely unchanged or only marginally increased (Figure S2A). To address whether the decrease in cysteine and asparagine/aspartate were detrimental to cells following proteasome inhibition, we examined whether supplementation with these amino acids affected cell viability of proteasome-inhibited cells. Amino acids were added after a transient treatment with the proteasome inhibitor to ensure that the amino acids did not interfere with the proteasome inhibitors. Addition of cysteine following treatment with MG-132 for 8 hr dramatically increased cell survival and markedly reduced apoptosis in a dose-dependent manner, in contrast to other amino acids, the reducing agent β-mercaptoethanol, or the antioxidant ascorbic acid (Figures S2B and S2C). Cysteine also markedly increased cell viability following Bortezomib treatment (Figure S2D). Asparagine further increased viability, when added together with cysteine following an 8 hr MG-132 treatment, whereas it only had a minor effect on its own (Figure 2A). It may not be a coincidence that both cysteine and asparagine are conditionally essential amino acids (Reeds, 2000), absent in the Dulbecco’s modified Eagle’s medium (DMEM). Although rescuing the viability of proteasome-inhibited cells, amino acid supplementation had no significant effect on the proliferation of untreated cells (Figure S2E) and neither cysteine nor asparagine detectably altered proteasome activity (Figure S2F). Thus, supplementation with the critical amino acids depleted upon proteasome inhibition rescued survival of ∼90% of proteasome-impaired cells, without rescuing proteasome activity.

To dissect the mechanism underlying the rescued viability of proteasome-inhibited cells by amino acid supplementation, we next focused on cysteine addition as it had a potent effect. We monitored the abundance of polyubiquitinated proteins as well as the proteasome reporter substrates Ub^G76V^-GFP, ZsGreen-ODC, and GFP-CL1 (Dantuma et al., 2000; Hoyt et al., 2005; Gilon et al., 1998; Bence et al., 2001). Although markedly increasing the survival of three different cell lines following proteasome inhibition (Figure S2G), addition of cysteine did not reduce the abundance of either polyubiquitinated proteins (Figure 2B) or the three different rapidly degraded, proteasome reporter-substrates Ub^G76V^-GFP, ZsGreen-ODC, and GFP-CL1 (Figure 2C). Similarly, cells accumulated indistinguishable levels of the proteasome reporter substrate ZsGreen-ODC when treated with MG-132 alone or together with 1 mM cysteine (Figure 2D). Addition of cysteine together with MG-132 also rescued the viability of proteasome-inhibited cells (Figure S2G). These results demonstrate that cysteine did not antagonize MG-132, while rescuing viability of cells following proteasome inhibition.

The proteasome activates the transcription regulator NF-κB by proteolytic cleavage of the precursor p105 into p50 (Palombella et al., 1994). Although rescuing cell viability (Figure 2A), cysteine supplementation did not reduce the impairment of the proteasome-mediated processing of the transcription regulator NF-κB, p105 into p50, in cells treated with MG-132 for 8 hr (Figure 2E). These results reveal that while rescuing from the lethality resulting from proteasome inhibition, supplementation with an amino acid selectively depleted following proteasome inhibition did not decrease the levels of polyubiquitinated proteins, proteasome reporter substrates and the impairment of NF-kB processing in proteasome-inhibited cells. Thus, cells can tolerate proteasome inhibition but not the resulting amino acid insufficiency.

### The Integrated Stress Response Is Induced by the Amino Acid Shortage Resulting from Proteasome Inhibition

**Figure 3:**
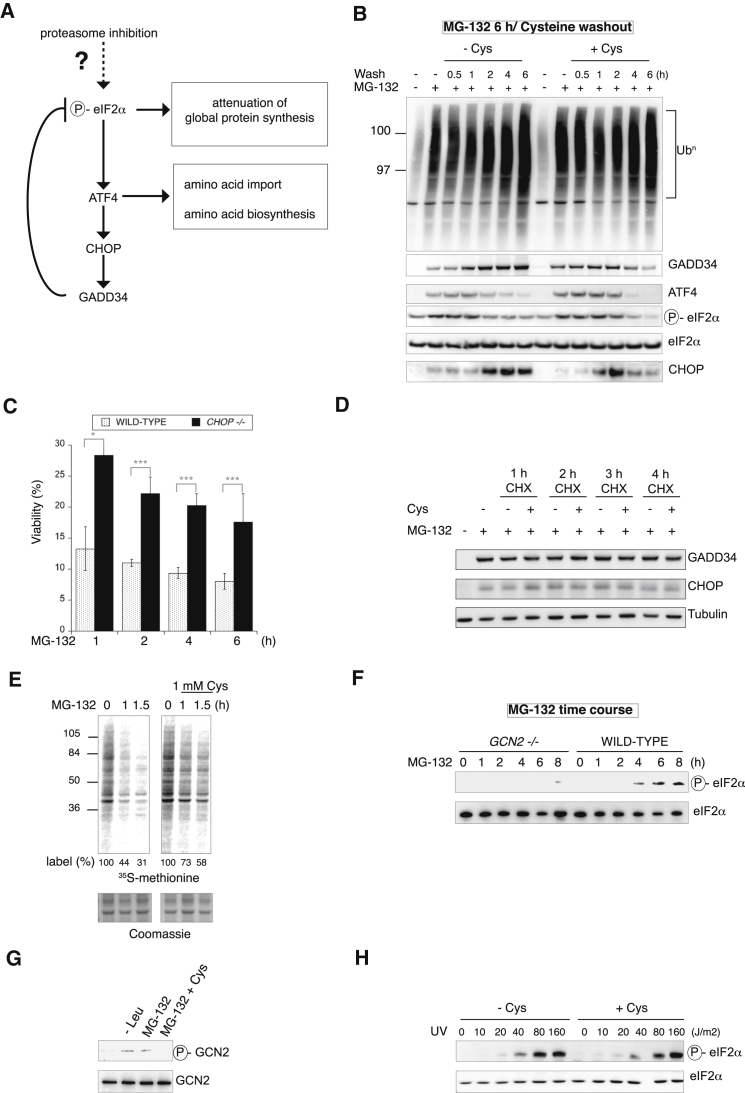
Amino Acid Supplementation Alleviates the Induction of the Integrated Stress Response upon Proteasome Inhibition (A) Scheme depicting the integrated stress response induction upon proteasome inhibition. The signal responsible for eIF2α phosphorylation (P-eIF2α) upon proteasome inhibition is unknown. (B) Polyubiquitin (Ub^n^), GADD34, ATF4, phosphorylated eIF2α (P-eIF2α), eIF2α, and CHOP immunoblots of lysates from NIH 3T3 cells treated with 10 μM MG-132 for 6 hr followed by a washout in the presence of 1 mM cysteine (Cys) for the indicated time. (C) Viability of wild-type and *CHOP−/−* mouse embryonic fibroblasts, assessed by the reduction of WST-8 into formazan, after 2 days of growth, following treatment with 10 μM MG-132 for the indicated time and a washout in regular medium. Data are means ± SD (n = 4), normalized to cell number. ^∗^p ≤ 0.01, ^∗∗∗^p ≤ 0.0001. (D) GADD34, CHOP, and tubulin immunoblots of lysates from NIH 3T3 cells treated with 10 μM MG-132 for 4 hr followed by a washout in the presence of cycloheximide (CHX, 100 μg/ml), with or without 1 mM Cys for the indicated time. (E) Autoradiogram of [^35^S]methionine-labeled proteins in lysates resolved by NuPage after a 8 min pulse labeling of exponentially growing cells cultured as indicated. Quantification are presented below each lane. Lower panel is a photomicrograph of the Coomassie-stained gel. (F) eIF2α immunoblots of lysates from *GCN2−/−* or wild-type mouse embryonic fibroblasts treated with 10 μM MG-132 for the indicated time. (G) Immunoblots of GCN2 immunoprecipitated from lysates of cells either left untreated, leucine-deprived (-Leu), or treated with 10 μM MG-132, with or without 1 mM Cys. The top panel was probed with antisera against the phosphorylated GCN2 and the bottom panel with an antibody recognizing both the phosphorylated and the nonphosphorylated GCN2. (H) Immunoblots of lysates from NIH 3T3 cells following a dose response analysis of UV irradiation, in the presence or in absence of 1 mM cysteine (Cys). Representative results of at least three independent experiments are shown. See also Figure S3.

Proteasome inhibition, like many different stresses, induces the integrated stress response (ISR) (Jiang and Wek, 2005). However, the signal responsible for the ISR induction in proteasome-inhibited cells is unknown. The ISR is an adaptive response to many forms of stresses, which converge into phosphorylating the α subunit of translation initiation factor 2 (eIF2α) thereby reprogramming protein synthesis. When eIF2α is phosphorylated, general protein synthesis is attenuated, whereas ATF4 is selectively translated (Harding et al., 2003). ATF4 is a transcription factor, which controls expression of genes involved in amino acid import and biosynthesis (Harding et al., 2003), as well as the transcription factor CHOP, which in turn induces GADD34 (Figure 3A). GADD34 is a regulatory subunit of the serine/threonine phosphatase PP1, which recruits PP1 in stressed cells to dephosphorylate eIF2α and terminates stress signaling (Novoa et al., 2003). Having found that proteasome inhibition caused a lethal shortage in both cysteine and asparagine, and because genes involved in the biosynthesis of cysteine and asparagine are prominent targets of the ISR (Harding et al., 2003), we examined whether amino acid scarcity may be the signal that induces the ISR in proteasome inhibited cells (Figure 3A). If so, amino acid supplementation should attenuate ISR signaling in response to proteasome inhibition. In the absence of MG-132, the ISR was not induced, confirming that untreated cells were not stressed (Figure 3B). As expected (Jiang and Wek, 2005), MG-132 induced the ISR, and this is manifested by the phosphorylation of eIF2α, expression of ATF4 and the pro-death proteins CHOP and GADD34 (Figure 3B). Strikingly, the levels of CHOP and GADD34 remained elevated in the MG-132 cells 6 hr after the removal of the proteasome inhibitor, in agreement with the finding that such cells were committed to die (Figure 2A). In contrast, in cells supplemented with cysteine, a marked decrease in the levels of CHOP and GADD34 was detected 4–6 hr following the cysteine washout (Figure 3B), in good agreement with the cytoprotective effect of cysteine upon proteasome inhibition (Figure 2A). When added together with MG-132, cysteine virtually abrogated the induction of the ISR resulting from proteasome inhibition, without altering the levels of polyubiquitinated proteins (Figure S3). This reveals that the amino acid shortage resulting from proteasome inhibition is the signal that induces the ISR in proteasome-inhibited cells.

To confirm that CHOP-mediated cell death following proteasome inhibition (Jiang and Wek, 2005), we monitored the survival of wild-type and *CHOP*−/− cells (Marciniak et al., 2004) following proteasome inhibition. Genetic ablation of *CHOP* markedly enhanced survival of proteasome-inhibited cells (Figure 3C). The decreased CHOP levels in NIH 3T3 cells supplemented with cysteine following proteasome inhibition could be due to an enhanced degradation or a decreased synthesis. We found that addition of cysteine did not rescue proteasomal degradation (Figures 2B–2E) and the stability of both GADD34 and CHOP were indistinguishable in cells that had been supplemented or not with cysteine, following proteasome inhibition (Figure 3D). Thus, the low levels of CHOP in proteasome-inhibited cells supplemented with cysteine did not reflect an increased degradation but rather, a decreased synthesis of this stress protein, as a result of the dephosphorylation of eIF2α following cysteine supplementation (Figure 2B).

The marked attenuation of eIF2α phosphorylation by cysteine supplementation in proteasome-inhibited cells suggested that the supply of cysteine might be rate-limiting for global protein synthesis in proteasome-inhibited cells. If so, cysteine supplementation may rescue global protein synthesis while attenuating translation of stressed proteins such as CHOP. We next tested this hypothesis. As expected (Jiang and Wek, 2005), proteasome inhibition caused a pronounced inhibition of protein synthesis (Figure 3E). Remarkably, addition of 1 mM cysteine largely prevented the translation attenuation resulting form proteasome inhibition (Figure 3E). GCN2 is the eIF2α kinase that mediates the ISR induction in response to amino acid deprivation (Harding et al., 2000; Zhang et al., 2002). Genetic ablation of *GCN2* virtually abolished eIF2α phosphorylation upon proteasome inhibition (Figure 3F). Furthermore, activated and phosphorylated GCN2 was detected in cells deprived of leucine or MG-132-treated cells, and addition of cysteine markedly reduced GCN2 phosphorylation upon proteasome inhibition (Figure 3G). This establishes that inhibition of proteasomal degradation causes a shortage of critical amino acids that induce the ISR via GCN2.

To confirm that the attenuation of eIF2α phosphorylation by cysteine in proteasome inhibited cells is due to the replenishment of this otherwise limiting amino acid, as opposed to a suppression of ISR signaling by an indirect effect, we tested whether cysteine affected eIF2α phosphorylation induced by ultraviolet (UV) irradiation, a different stress than amino acid limitation (Deng et al., 2002). We found that the induction of eIF2α phosphorylation by UV was not reduced by supplementation with cysteine (Figure 3H). Thus, cysteine supplementation selectively alleviates eIF2α phosphorylation in proteasome-inhibited cells, but not in UV-irradiated cells.

### Amino Acid Scarcity in Proteasome-Inhibited Cells Signals the Induction of Autophagy

**Figure 4:**
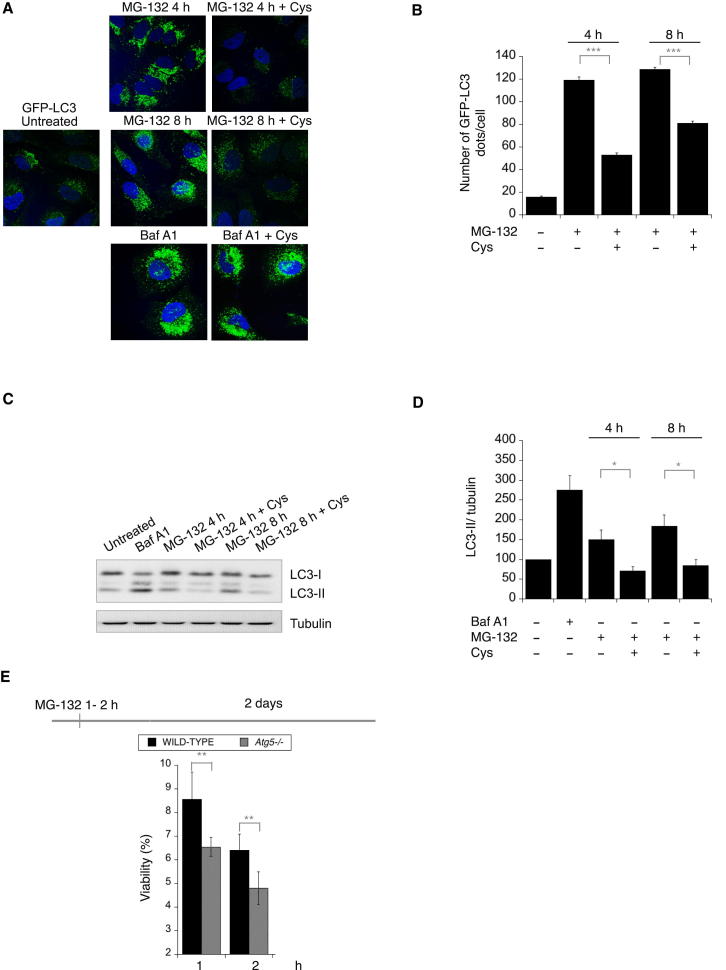
Amino Acid Supplementation Suppresses Autophagy Resulting from Proteasome Inhibition (A) Confocal micrographs of HeLa cells stably expressing GFP-LC3 (Thurston et al., 2009) either untreated or following 10 μM MG-132 treatment, with or without 1 mM Cys, where indicated. Nuclei were stained with H33258. (B) Quantification of GFP-LC3 dots in at least 100 cells exposed to the indicated treatment. Data are means ± SEM. ^∗∗∗^p ≤ 0.0001. (C) LC3 and tubulin immunoblots of lysates from cells treated with 100 nM Bafilomycin A1 (Baf A1) for 15 hr, or 10 μM MG-132 alone or together with 1 mM Cys for the indicated time. (D) Quantification of the LC3-II/tubulin ratio in three independent experiments. Values were normalized to untreated cells. Data are means ± SEM. ^∗^p ≤ 0.05. (E) Viability of wild-type and *Atg5−/−* mouse embryonic fibroblasts, assessed by the reduction of WST-8 into formazan, after 2 days of growth, following treatment with 10 μM MG-132 for the indicated time and a washout in regular medium. Data are means ± SD (n = 4), normalized to cell number. ^∗∗^p ≤ 0.001. n.s., not significant. See also Figure S4.

Although polyubiquitination is the canonical signal to target proteins for proteasomal degradation, recent studies have revealed that polyubiquitinated proteins can also be degraded by autophagy (Kirkin et al., 2009). Autophagy is also induced when proteasomal degradation is inhibited (Rideout et al., 2004; Iwata et al., 2005) and alleviates the toxicity resulting from proteasome inhibition (Korolchuk et al., 2010; Pandey et al., 2007). However, the mechanisms underlying autophagy induction when proteasomal degradation is compromised are unknown. As expected (Korolchuk et al., 2010), MG-132 increased the number of GFP-LC3 labeled autophagosomes in HeLa cells stably expressing GFP-LC3 (Figures 4A and 4B). Strikingly, cysteine supplementation markedly decreased the number of fluorescent puncta in proteasome-inhibited cells (Figures 4A and 4B). In contrast, supplementation with other amino acids, reducing agents or antioxidants did not noticeably reduced the number of GFP-LC3 puncta in proteasome inhibited cells (Figure S4). Cysteine is not a generic suppressor of autophagosome accumulation because cysteine did not decrease the number of GFP-LC3 puncta that accumulated in cells treated with Bafilomycin A1 (Figures 4A and 4B), an inhibitor of autophagosome-lysosome fusion (Mizushima et al., 2010). MG-132 treatment also increased the levels of LC3-II (Figures 4C and 4D) but this was markedly decreased in the presence of cysteine (Figures 4C and 4D). These results establish that the decrease in a critical amino acid is the signal that induces autophagy when proteasomal degradation is inhibited. To assess whether induction of autophagy contributed to protect cells from proteasome-inhibition, we compared the survival of wild-type or *Atg5−/−* cells (Kuma et al., 2004) following proteasome inhibition. We found that *Atg5−/−* cells were significantly more sensitive to MG-132 treatment and this was manifest already following 1 hr of proteasome inhibition (Figure 4E).

**Figure 5:**
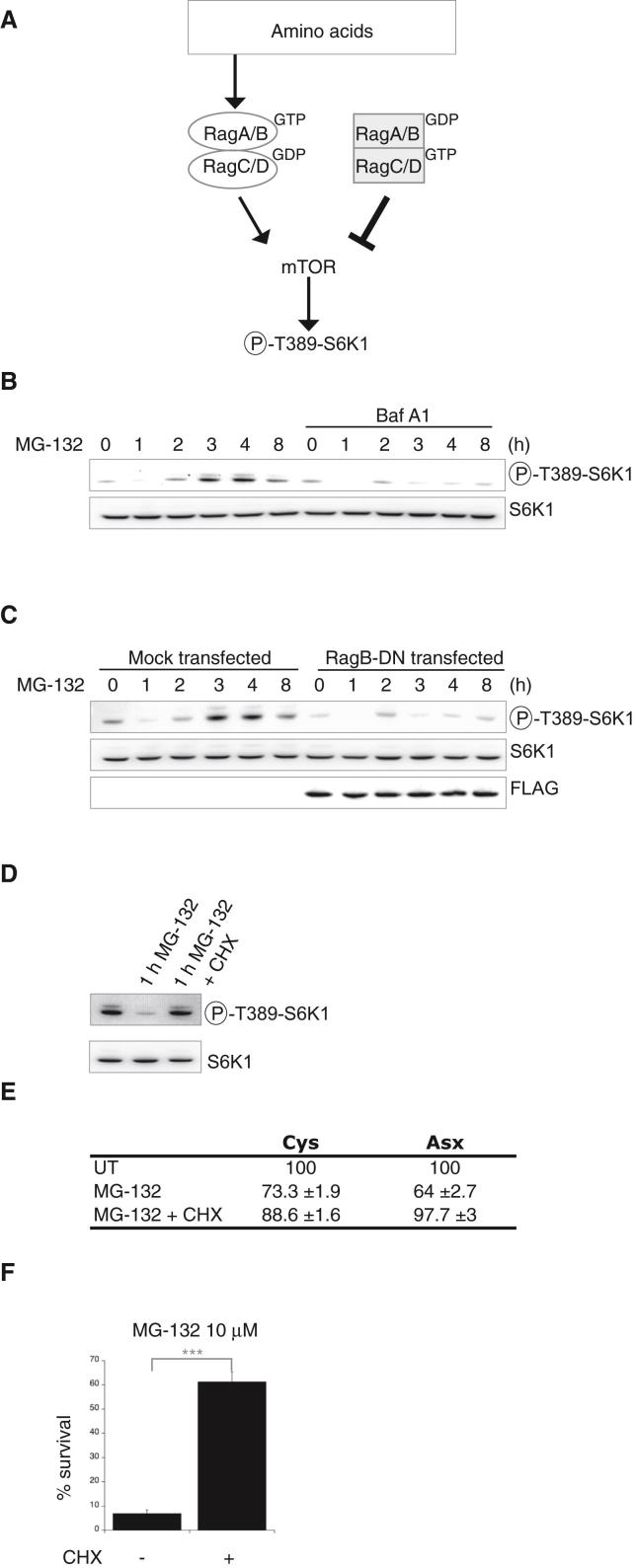
Autophagy and Rag GTPases-Dependent Amino Acid Signaling to mTOR upon Proteasome Inhibition (A) Model for amino acid signaling to mTOR by the Rag GTPases (RagA/B^GTP^-RagC/D^GDP^). Amino acid sensing by the Rag GTPases is blocked by dominant negative mutants (RagB^GDP^-RagC^GTP^) (Sancak et al., 2008). (B) Immunoblots of lysates from 293T cells exposed to 10 μM MG-132 treatment revealed with phospho-T398-S6K1 or S6K1 antibodies. Cells were either left untreated or treated with 100 nM Baf A1 for the indicated time. (C) Immunoblots with phospho-T398-S6K1 or S6K1 antibodies of lysates of cells mock transfected or transfected with dominant negative forms of RagB (RagB-DN, FLAG-tagged). (D) Immunoblots with phospho-T398-S6K1 or S6K1 antibodies of lysates of 293T cells either left untreated or treated with 10 μM MG-132 with or without 100 μM cycloheximide (CHX) for 1 hr. (E) Changes in the content of cellular free cysteine (Cys) or asparagine/aspartate (Asx) in cells treated with 10 μM MG-132 with or without 50 μM cycloheximide (CHX) for 4 hr. (F) Viability of NIH 3T3 cells, assessed by the reduction of WST-8 into formazan, after 2 days of growth, following treatment with 10 μM MG-132 with or without 50 μM cycloheximide (CHX) for 8 hr. Data are means ± SD (n = 4), normalized to cell number. ^∗∗∗^p ≤ 0.0001. Representative results of at least three independent experiments are shown.

The mammalian target of rapamycin (mTOR) signaling pathway senses and responds to amino acid availability to modulate protein synthesis via the phosphorylation of substrates such as S6 kinase 1 (S6K1) (Ma and Blenis, 2009) (Figure 5A). To evaluate whether autophagy contributed to replenish the pool of intracellular amino acids upon proteasome inhibition, we measured the levels of phosphorylation of threonine 389 of the mTORC1 substrate S6K1 (Ma and Blenis, 2009). In agreement with the decrease in critical amino acids following proteasome inhibition, S6K1 phosphorylation decreased 1 hr after MG-132 addition (Figure 5B). Four hours following MG-132 addition, S6K1 phosphorylation increased (Figure 5B). This increase was abrogated by Bafilomycin A1, indicating that it was dependent on autophagic degradation (Figure 5B). Amino acid sensing by mTOR is mediated by the Rag GTPases and dominant negative mutants of the Rag GTPases abrogate mTOR stimulation by amino acids (Sancak et al., 2008). Strikingly, overexpression of dominant negative mutants of the Rag GTPases that prevent amino acid sensing by mTORC1 (Figure 5A) also abolished the increased phosphorylation of S6K1 that followed proteasome inhibition (Figure 5C). These results reveal that amino acid scarcity is the signal that induces autophagy when the proteasome is inhibited, in an attempt to rescue amino acid homeostasis.

This model predicts that blocking amino acid consumption by inhibiting protein synthesis should recapitulate the rescuing effects of amino acid supplementation in proteasome inhibited cells. Indeed, we found that addition of cycloheximide together with MG-132 prevented the decreased S6K1 phosphorylation resulting from treatment with MG-132 for 1 hr (Figure 5D). Consistently, the decrease in cysteine and asparagine/aspartate resulting from proteasome inhibition was markedly attenuated in cells cotreated with cycloheximide (Figure 5E). Strikingly, cycloheximide protected cells from the lethality resulting from proteasome inhibition (Figure 5F). The rescuing effect of cycloheximide in proteasome-inhibited cells was selective because cycloheximide increased the lethality of cells exposed to UV irradiation (data not shown). Thus, inhibition of protein synthesis markedly attenuates the depletion of critical amino acid following proteasome inhibition as well as the lethality resulting from proteasome inhibition.

### An Amino Acid Imbalance Underlies the Lethality Resulting from Proteasome Inhibition in *Drosophila*

**Figure 6:**
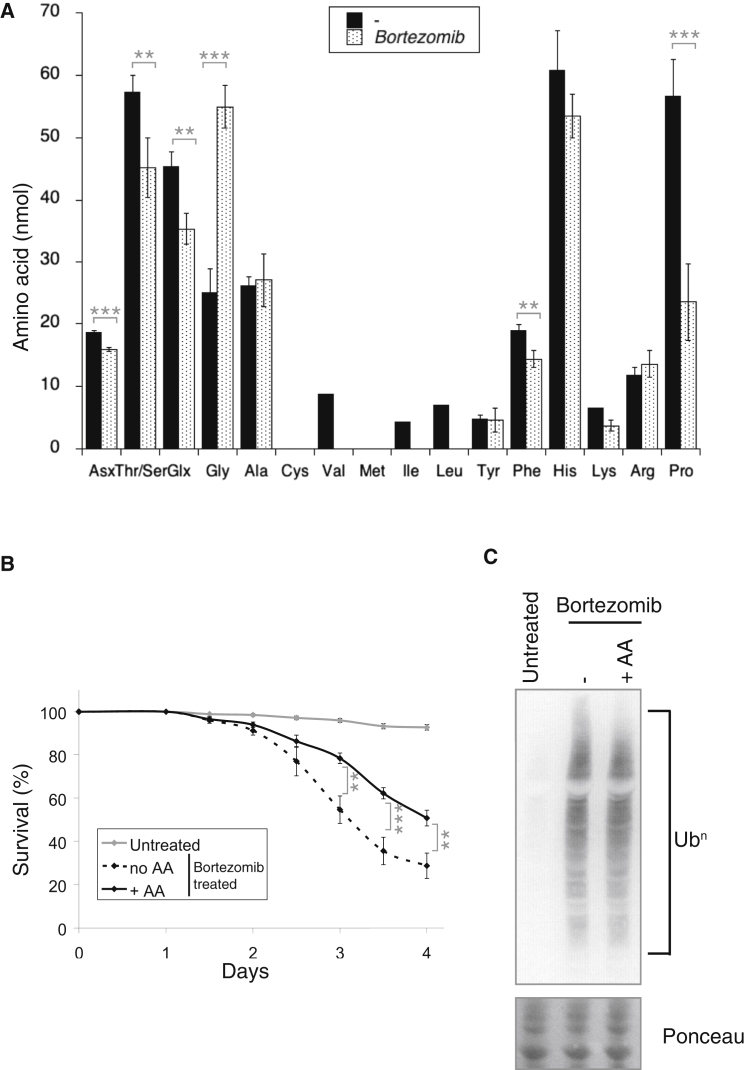
Amino Acid Imbalance Mediates the Lethality Resulting from Proteasome Inhibition in *Drosophila* (A) Changes in the content of free amino acids in *Drosophila* treated with or without 50 μM Bortezomib for 3 days. Asx, asparagine/aspartate; Glx, glutamine/glutamate. Data are means ± SEM (30 flies per conditions, from six independent experiments). ^∗∗^p ≤ 0.005; ^∗∗∗^p ≤ 0.001. (B) Survival of *Drosophila* either untreated or treated with 50 μM Bortezomib, with or without additional amino acids (AA), at the concentration used in Grandison et al. (2009). Data are means ± SEM (400 flies per condition, from four independent experiments). ^∗∗^p ≤ 0.005; ^∗∗∗^p ≤ 0.001. (C) Polyubiquitin (Ub^n^) immunoblots from lysates of flies treated with 50 μM Bortezomib and additional amino acids, where indicated. Ponceau staining of the membrane. Representative results of at least three independent experiments are shown.

We next assessed whether proteasome inhibition had detrimental consequences on amino acid homeostasis in an organismal context. We exposed *Drosophila* to proteasome inhibitors and monitored amino acid levels. Bortezomib significantly reduced the levels of asparagine/aspartate, threonine/serine, glutamine/glutamate, phenylalanine, and proline in *Drosophila* (Figure 6A). Bortezomib also increased the levels of glycine (Figure 6A), similar to prolonged starvation in humans (Felig et al., 1969). As expected (Vernace et al., 2007), exposure of flies to Bortezomib was lethal (Figure 6B). Amino acid supplementation markedly increased survival of proteasome-inhibited flies (Figure 6B), without detectable changes in the levels of proteasome substrates (Figure 6C). Thus, proteasome inhibition caused an intolerable amino acid imbalance in flies and the resulting lethality was rescued upon supplementation with amino acids.

## Discussion

**Figure 7:**
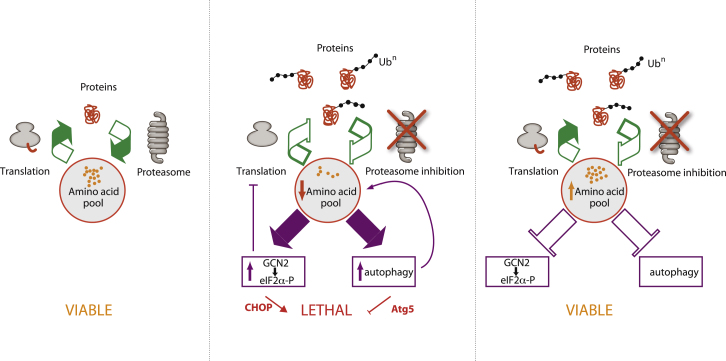
Proteasome Inhibition Causes an Intolerable Amino Acid Imbalance and the Resulting Lethality Is Rescued upon Supplementation with Amino Acids The amino acid shortage resulting from proteasome inhibition is the signal that induces eIF2α phosphorylation and autophagy, in a vain attempt to replenish to pool of amino acid and sustain vital protein synthesis rates. In yeast, mammalian cells, and *Drosophila*, the lethality resulting from proteasome inhibition is rescued upon amino acid supplementation. This rescuing effect occurs without a detectable decrease in the levels of proteasome substrates in proteasome-inhibited cells.

Here, we show that the detrimental consequences of proteasome inhibition are largely abrogated by supplementation with amino acids in yeast, mammalian cells, and *Drosophila* (Figure 7). Strikingly, the rescuing effect of amino acids occurs without reducing the accumulation of proteasome substrates. These findings reveal that cells can survive with the protein waste they accumulate when the proteasome is inhibited but the resulting amino acid deficiency is lethal. Thus, the proteasome substrates accumulating upon proteasome inhibition appear detrimental to cells as they sequester a pool of critical amino acids that would otherwise be recycled.

Although abrogating death of proteasome-inhibited cells, amino acid supplementation does not abrogate the requirement for proteasomal degradation for regulatory functions such as cell division. The amino acid scarcity resulting from proteasome inhibition induces the classical set of responses to amino acid starvation, in an vain attempt to sustain amino acid homeostasis. Although it had been previously reported that proteasome inhibition, like many different stresses, induces eIF2α phosphorylation and that the resulting translation attenuation is abrogated by genetic impairment of eIF2α phosphorylation (Jiang and Wek, 2005), the underlying mechanism was unknown. We have shown here that the amino acid decrease following proteasome inhibition is the signal that activates GCN2 and induces eIF2α phosphorylation, leading to attenuation of protein synthesis (Figure 3). Cysteine is the least abundant cellular amino acid and we found that cysteine scarcity following proteasome inhibition impairs protein synthesis. Upon persistent inhibition of the proteasome and the subsequent amino acid insufficiency, the ISR is chronically activated, thereby leading to the expression of the pro-apoptotic protein CHOP (Figure 3B). Supplementation with a critical amino acid, depleted upon proteasome inhibition, virtually suppresses ISR signaling and cell death (Figure 3B), similar to genetic ablation of *CHOP* (Figure 3C) or impairment of eIF2α phosphorylation, which reduces death following proteasome inhibition (Jiang and Wek, 2005).

The mechanisms underlying the crosstalk between autophagic and proteasomal degradation have remained elusive. In this study, we provide the missing link between autophagy induction and proteasome inhibition. We show that the signal responsible for autophagy induction upon proteasome inhibition is the resulting amino acid scarcity, the canonical inducer of autophagy. Autophagy aims at protecting cells from the deleterious consequences of proteasome inhibition, as cells lacking Atg5, a key autophagy component are more sensitive to proteasome inhibition. Both the ISR and autophagy are involved as early as 1 hr following proteasome inhibition to adapt cells to the resulting changes. Our work shows that autophagy and proteasomal degradation act in a concerted manner to adjust the supply of amino acids to the cellular needs. Following proteasome inhibition, autophagy attempts to restore amino acid homeostasis, as manifested by an increased S6K1 phosphorylation via the canonical, Rag GTPases-dependent amino acid signaling pathway to mTOR. The findings reported here reveal how changes in amino acid homeostasis are integrated with protein metabolism and cell death (Figure 7).

We found here that the deleterious consequences of proteasome inhibition are rescued upon amino acid supplementation in yeast, mammalian cells, and *Drosophila*. These findings establish the importance of proteasomal degradation in recycling amino acids for normal cell and organismal function. There is a qualitative difference in the required amino acids in the three different systems that is not surprising as yeast, mammalian cells, and *Drosophila* have different properties and requirements. Yeast cells required a broad range of amino acids to survive proteasome inhibition, perhaps because they have higher metabolic needs, as they divide more rapidly than mammalian cells. Upon proteasome inhibition, mammalian cells had a prominent need for cysteine and asparagine and we show that the decrease in cysteine and asparagine/aspartate were largely prevented by blocking protein synthesis in proteasome-inhibited cells (Figure 5E). Notably, enzymes required for the metabolism of cysteine and asparagine, two conditionally essential amino acids, are under the control of the ISR, thus underscoring an evolutionary pressure to maintain the levels of asparagine and cysteine in the cell. In addition, the importance of asparagine starvation has been previously recognized and is the basis for the use of L-asparaginase for the treatment of acute lymphoblastic leukemia (Müller and Boos, 1998).

The proteasome inhibitor Bortezomib is used in cancer therapy but it is limited to the treatment of multiple myeloma and restricted by adverse effects, such as peripheral neuropathy (Richardson et al., 2003). So far, the mechanisms by which proteasome inhibitors kill cells have remained elusive. Failure of amino acid homeostasis not only explains the lethality resulting from proteasome inhibition but also provides a foundation for the development of novel cancer therapies.

## Experimental Procedures

### Mammalian Cell Culture

NIH 3T3, 293T (ATCC), 293T cells stably expressing ZsGreen-ODC (ZsProsensor-1 cells, Clontech), and HeLa cells were maintained at 37°C in DMEM supplemented with 10% fetal bovine serum (FBS), penicillin, and streptomycin. MEFs cells were cultured in DMEM supplemented with penicillin, streptomycin, glutamine, 1× nonessential amino acids (Sigma), sodium pyruvate, and 10% FBS.

### Assessment of Cell Viability

Cells were plated (day 0) in DMEM supplemented with 10% FBS bovine serum in 24-well plates (Nunc), at a density of 8,000 cells/ml. The next day (day 1), media were replaced with DMEM supplemented with 0.5% FBS. On day 2, cells were treated with proteasome inhibitors for the indicated period of time. Media containing proteasome inhibitors were replaced with DMEM supplemented with 0.5% FBS and the indicated additives. Four wells per condition were used. On day 3, media were replaced with DMEM supplemented with 10% FBS. Cells were kept for 2–3 days as indicated. Cell viability was assessed by measuring the reduction of WST-8 [2-(2-methoxy-4-nitrophenyl)-3-(4-nitrophenyl)-5-(2,4-disulfophenyl)-2H-tetrazolium] into formazan using Cell Viability Counting Kit-8 (Dojindo) according to the supplier’s recommendation.

### Flow Cytometry and Confocal Microscopy

Flow cytometry analyses were performed on a FACSCalibur flow cytometer (BD Biosciences) and analyzed with FlowJo and confocal microscopy was carried out as described in Münch et al. (2011).

### Immunoblot Analyses and Assessment of Translation Rates

Cell lysis, immunoblots, and measurement of translation rates were performed as described in Tsaytler et al. (2011). Note that cells were not starved in methionine-free media before labeling with ^35^S-methionine for monitoring protein synthesis.

### Yeast Strains, Media, and Culture

*Cim3-1* or *pre1-1* and their respective isogenic wild-type strains (Ghislain et al., 1993; Heinemeyer et al., 1991) and *ndc80-1* (Wigge et al., 1998) were grown in YPD medium using standard techniques, as in Dehay and Bertolotti (2006).

### Amino Acid Analyses

Amino acid analysis from yeast cells and mammalian cells were performed as described, respectively (Onodera and Ohsumi, 2005; Vabulas and Hartl, 2005).

### Flies

Wild-type Oregon R flies were reared on standard food. Amino acids were extracted in 600 μl methanol from 5 frozen female flies per condition, as in Hayward et al. (1993). The suspension was centrifuged and the supernatant collected, vacuum-dried, and analyzed. Some amino acids were below detection levels in such analyses. For survival experiments, 20 flies, both females and males, were kept in vials lined with 3 mm filter paper and fed a solution of 5% sucrose with either vehicle (0.5% DMSO, control) or with Bortezomib (50 μM in DMSO), for the indicated time, as in Vernace et al. (2007). Under the same conditions MG-132 (50 μM in DMSO) was not toxic. In Figure 6B, 1.5 ml of 5% sucrose with or without amino acids at the concentration used in Grandison et al. (2009) was added together with the proteasome inhibitor. Proteins were extracted from five frozen female flies per conditions in 250 μl boiling Laemmli buffer supplemented with 10 mM NEM and 50 mM DTT.

### Statistical Analysis

Data are presented as means and SD or SEM, as indicated. Where indicated, two groups were compared with a two-tailed nonpaired Student’s t test.

## Supplemental Information

Document S1. Figures S1–S4 and Supplemental Experimental Procedures
