# Supplementary material for: Failure of Amino Acid Homeostasis Causes Cell Death following Proteasome Inhibition
Source: Mol Cell. 2012 Oct 26;48(2):242–53. doi: 10.1016/j.molcel.2012.08.003 (PMC3482661; doi:10.1016/j.molcel.2012.08.003)
Supplement: Document S1. Figures S1–S4 and Supplemental Experimental Procedures [file mmc1.pdf]

Molecular Cell, Volume *48*

Supplemental Information

Failure of Amino Acid Homeostasis Causes Cell Death following Proteasome Inhibition

Amila Suraweera, Christian Münch, Ariane Hanssum, and Anne Bertolotti

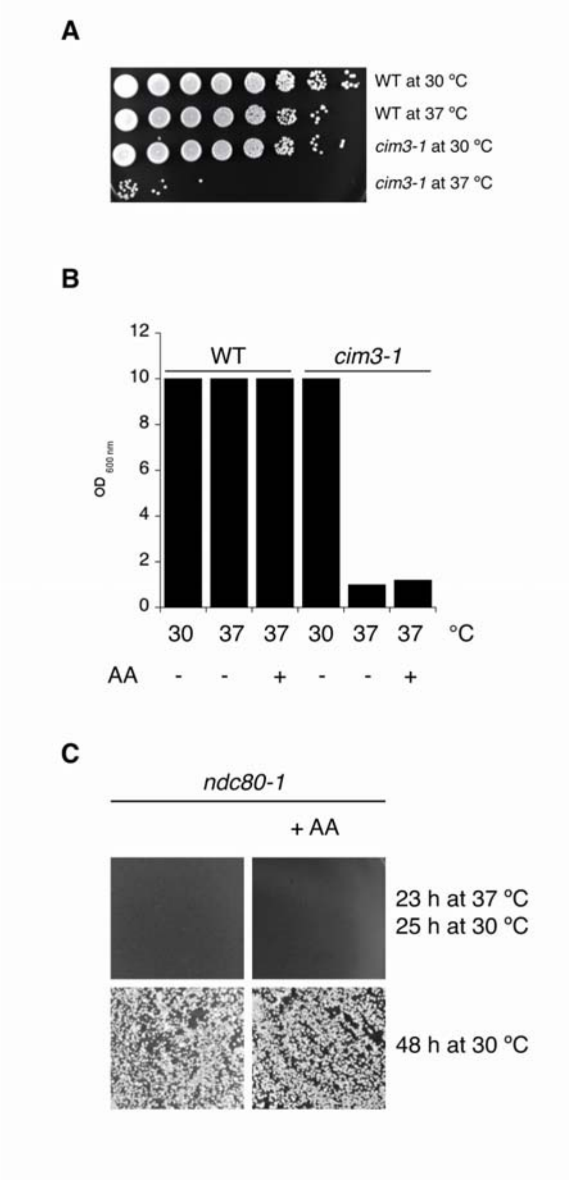

Figure S1. Thermosensitivity of the Yeast *cim3-1* and *ndc80-1* Cells

(A) Serial dilutions (1/10) of *cim3-1* and isogenic wild-type strains (WT) grown for 20 h in liquid cultures at the indicated temperatures and spotted on rich medium and incubated at 30 ºC for 2 days.

(B) Optical density of yeast cells (OD_{600nm}) of the indicated genotypes grown for 21 h at the indicated temperature, with or without casamino acids (AA, 40 g/l).

(C) *ndc80-1* cells were plated (2000 cells/plate) on rich medium with or without casamino acids (AA, 40 g/l) and grown at the indicated temperatures for the indicated time. Representative results of at least 3 independent experiments are shown.

A

Amino acid	nmol/10⁶ cells	% Untreated	
		MG-132	Bortezomib
Asx	27.3 ± 0.26	68.11 ± 1.75	78.95 ± 1.26
Thr/Ser	54.56 ± 1.72	113.76 ± 4.22	114.43 ± 5.65
Glx	55.76 ± 1.87	95.18 ± 4.38	107.86 ± 1.76
Gly	68.89 ± 2.30	99.73 ± 3.5	108.69 ± 2.28
Ala	7.17 ± 0.09	120.23 ± 4.86	152.37 ± 1.68
Cys	1.29 ± 0.03	75.85 ± 5.34	76.22 ± 6.05
Val	9.88 ± 0.21	124.95 ± 0.50	108.15 ± 3.67
Met	2.84 ± 0.24	109.04 ± 5.58	94.26 ± 10.44
Ile	9.16 ± 0.08	125.10 ± 3.29	108.94 ± 1.97
Leu	9.41 ± 0.24	126.33 ± 4.32	109.75 ± 3.47
Tyr	5.87 ± 0.20	121.95 ± 1.40	107.31 ± 2.67
Phe	7.89 ± 0.17	103.67 ± 3.14	105.73 ± 5.24
His	5.91 ± 1.44	104.08 ± 1.01	105.72 ± 9.29
Lys	4.48 ± 0.18	96.74 ± 12.14	102.72 ± 7.01
Arg	4.14 ± 0.07	89.46 ± 7.53	128.14 ± 8.36
Pro	10.47 ± 1.02	101.89 ± 1.79	117.94 ± 15.84

B

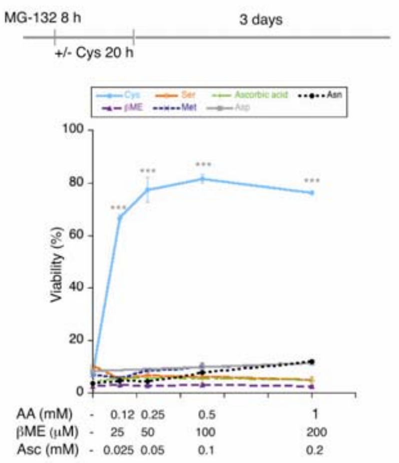

C

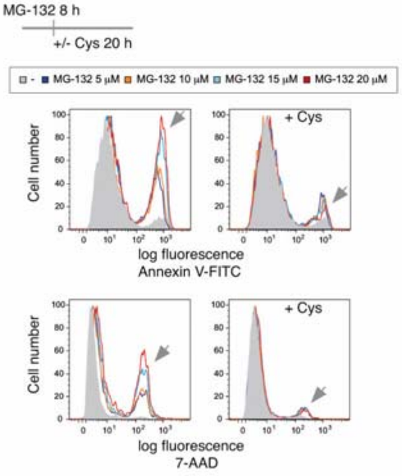

D

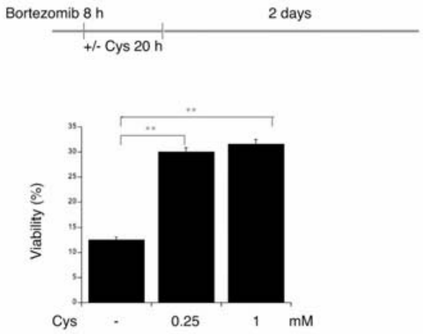

E

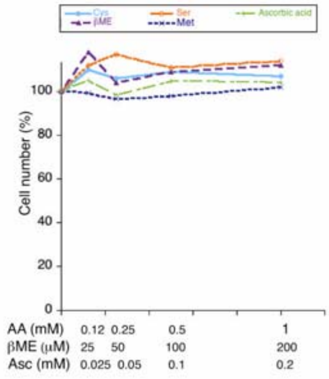

F

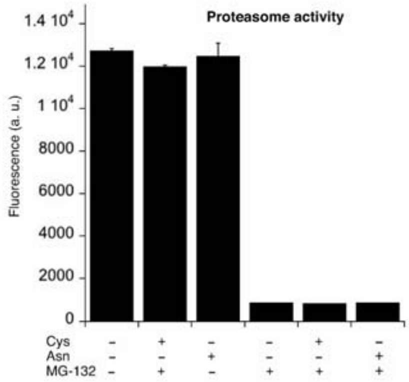

G

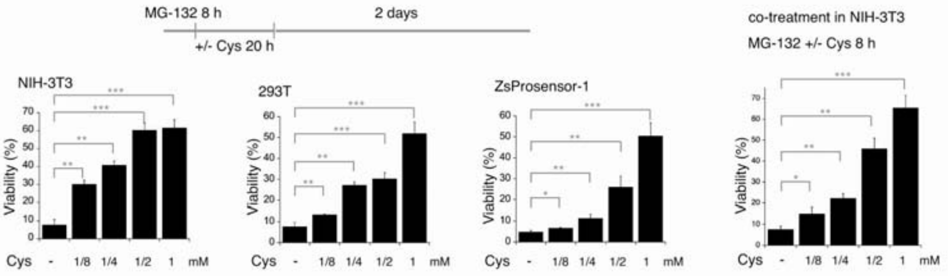

Figure S2. Cell Death Following Proteasome Inhibition Arises from Failure of Amino Acid Homeostasis

(A) Changes in the content of free amino acids in NIH-3T3 cells treated with 25 μM MG-132 or Bortezomib for 4 h. Data are means ± s.d. (n=3). Right columns: Values were normalized to untreated cells. Asx: asparagine/aspartate. Glx: glutamine/glutamate.

(B) Viability of NIH-3T3 cells, assessed by the ability to reduce WST-8 into formazan, following 5-20 μM MG-132 treatments and a 20 h washout in the presence or absence of the indicated amino acids (AA), the reducing agent β-mercaptoethanol (βME) or the antioxidant ascorbic acid (Asc). Data are means ± s.d. (*n*=4). ***$P \leq 0.0001$.

(C) Apoptosis monitored by Annexin V-FITC or 7-AAD staining and flow cytometry of NIH-3T3 cells after treatment with 10 μM MG-132 for 8 h followed by a 20 h washout in the presence or absence of 1 mM cysteine (Cys). Arrows point to apoptotic cell population.

(D) Viability of NIH-3T3 cells treated with 10 μM Bortezomib for 8 h, followed by a 20 h washout with or without Cys. Data are means ± s.d. (*n*=4). **$P \leq 0.001$.

(E) Assessment of the number of NIH-3T3 cells supplemented with the indicated additives for 20 h. Data are means.

(F) Proteasome activity is not altered by cysteine or asparagine. The chymotrypsin-like activity of rabbit 26S proteasome was assessed in the presence or in absence of Cys (1mM) or Asn (10mM) and MG-132 (10 μm) where indicated. Fluorescence arbitrary units (a.u.). Data are means ± s.d. (*n*=3).

(G) Viability of NIH-3T3, 293T, or ZsProsensor-1 cells subjected to treatment with 10 μM MG-132 for 8 h, followed by a 20 h washout with or without 1 mM cysteine (Cys) or co-treatment with MG-132 in the presence or absence of 1 mM Cys for 8 h. Data are means ± s.d. (*n*=4). *$P \leq 0.01$, **$P \leq 0.001$ and ***$P \leq 0.0001$.

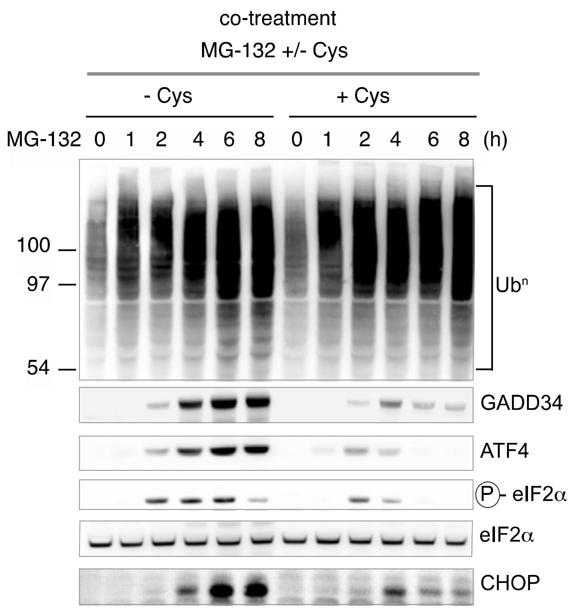

Figure S3. Amino Acid Supplementation Alleviates the Induction of the Integrated Stress Response (ISR) upon Proteasome Inhibition

Polyubiquitin (Ub^n), GADD34, ATF4, phosphorylated eIF2α (P-eIF2α), eIF2α and CHOP immunoblots of lysates from NIH-3T3 cells treated with 10 μM MG-132 in the presence or absence of 1 mM cysteine (Cys) for the indicated time.

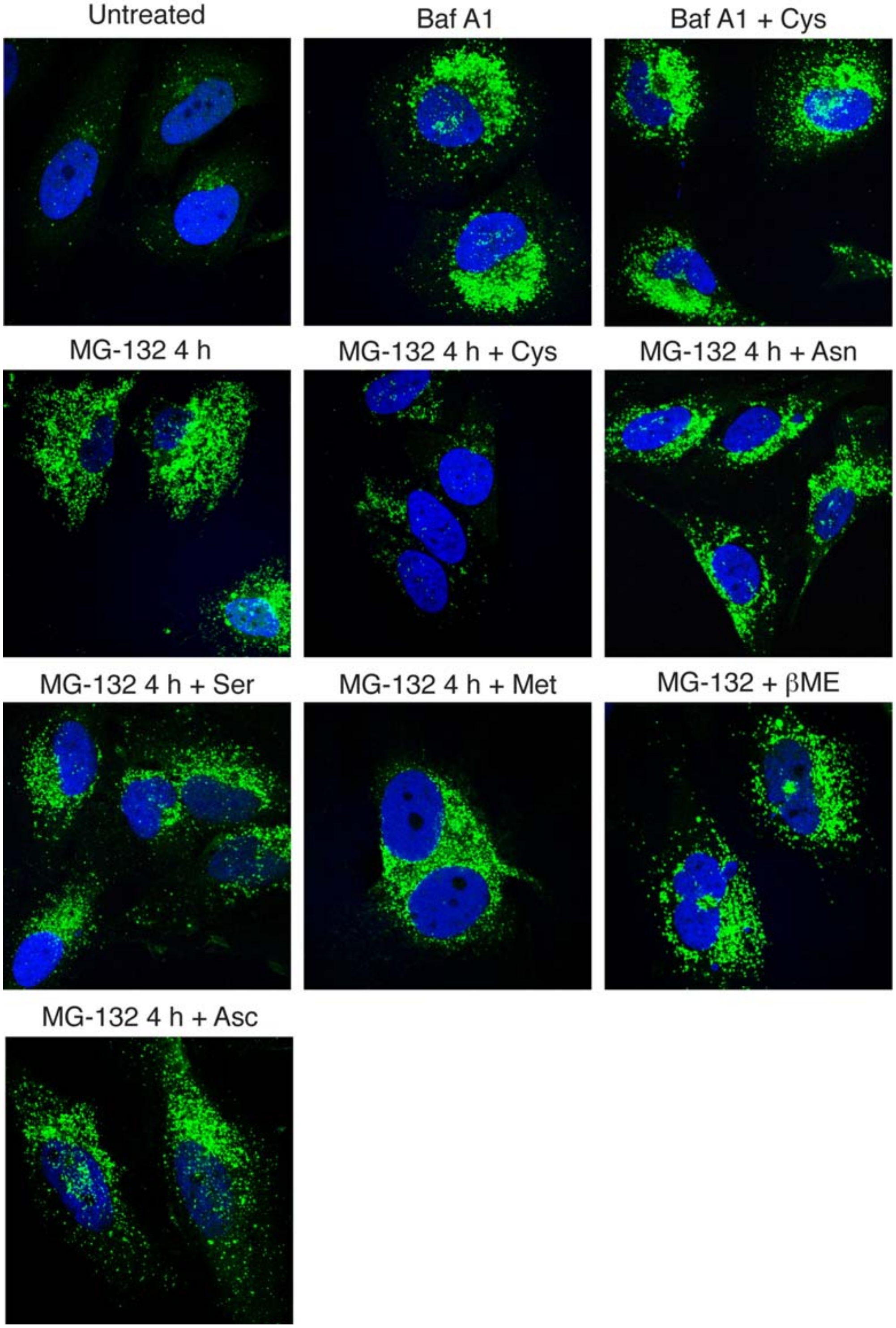
Untreated
Baf A1
Baf A1 + Cys
MG-132 4 h
MG-132 4 h + Cys
MG-132 4 h + Asn
MG-132 4 h + Ser
MG-132 4 h + Met
MG-132 + βME
MG-132 4 h + Asc

Figure S4. Addition of Cys Prevents Induction of Autophagy upon Proteasome Inhibition

Confocal micrographs of HeLa cells stably expressing GFP-LC3 (Thurston et al., 2009) either untreated or following treatment for 4 h with 10 μM MG-132 or 100 nM Bafilomycin A1 (Baf A1) for 15 h, with or without 1 mM of the indicated amino acid, 0.2 mM ascorbic acid (Asc) or 200 μM β-mercaptoethanol (βME). Nuclei were stained with H33258.

Supplemental Experimental Procedures

Mammalian cells treatments

MG-132 (Calbiochem) and Bortezomib (LC-laboratories) were solubilized in DMSO and kept at -80 ºC in DMSO for up to 2 weeks. Amino acids, β-mercaptoethanol, ascorbic acid and Bafilomycin A1 were from Sigma-Aldrich and prepared freshly. Cycloheximide was from Acros Organics. 48 h prior to the experiment, cells were plated at a density of 30 000 cells/ml for immunoblots or flow cytometry analyses. 24 h prior to treatment with proteasome inhibitors, cells were transferred to DMEM supplemented with 0.5% FBS. In low serum, cell proliferation during the treatments with proteasome inhibitors and additional amino acids is negligible. 293T cells were transfected with NF-κB expression plasmid, Addgene plasmid 23288 (Beraud et al., 1994) or with RagB dominant negative ($RagB^{GDP}$=T54 and $RagC^{GTP}$=Q120L) (Sancak et al., 2008; Narita et al., 2011) using Lipofectamine 2000 or the calcium phosphate method as described in (Rousseau et al., 2009). Cells were treated with proteasome inhibitors 40 h after transfection. For UV irradiation, 85% confluent NIH-3T3 cells in 10 cm plates were washed twice with phosphate-buffered saline (PBS) and irradiated in 1 ml PBS with the UV doses indicated (254 nm, UV stratalinker 2400, Stratagene). Cells were then allowed to recover in complete media, with or without cysteine, for 30 minutes before cell lysis and subsequent immunoblot analyses.

Antibodies

CLB2 (Ghislain et al., 1993) (1/100 dilution), phospho-eIf2α [pS^{52}] (44-728G; Invitrogen; 1/1000 dilution), eIF2α (ab5369; Abcam, 1/1000 dilution), ATF4/CREB-2 (sc-200; Santa Cruz Biotechnology, 1/500 dilution), GADD34/PPP1R15A (10449-1-AP; ProteinTech Group, 1/1000 dilution), CHOP (MA1-250; ABR Affinity BioReagents, 1/1000 dilution), α-tubulin (T5168; Sigma-Aldrich, 1/4000 dilution), polyubiquitin (FK2, MBL International, 1/200 dilution), NF-κB (ab7971, Abcam, 1/400 dilution), LC3 (PM036, MBL, 1/1000 dilution), phospho-S6K1 T389 (9205; Cell Signaling, 1/500 dilution), S6K1 (9202; Cell Signaling, 1/500 dilution) and FLAG antibody (F3165; Sigma, 1/1000 dilution). Immunoprecipitation and detection of GCN2 were done as described (Harding et al., 2000).

Amino acid analyses

Yeast cells (10 units, OD_{600}) were washed twice with distilled water, resuspended in 500 μl of distilled water, and boiled for 15 min, as described (Onodera and Ohsumi, 2005). The suspension was centrifuged and the supernatant was collected and vaccum-dried. One million NIH-3T3 cells were either left untreated or treated with the indicated proteasome inhibitors for the indicated time. Whole-cell free amino acids were collected from mammalian cells as described (Vabulas and Hartl, 2005). Cells were rapidly washed 3 times in ice cold PBS and lyzed in 10% TCA. Soluble material was vacuum-dried and subjected to analysis. Proteins were quantified (Bio-Rad protein assay) in the solubilized pellets to ensure that the samples had equal protein concentration. The samples were next dissolved in 500 μl of Sodium citrate loading buffer pH 2.2 and 50 μl was injected for analysis (Pharmacia Biochrom 20), using an *in situ* derivatization with ninhydrin followed by a separation, identification and quantitation by ion-exchange chromatography. The instrument was calibrated with 100 nmoles standard amino acid mixture and 50 nmoles of cysteine. Note that tryptophan is not detectable in such analyses.

Assessment of yeast viability

To assess growth phenotypes, the indicated strains were grown in liquid YPD at 30 ºC up to mid-log phase. Thereafter, cell density was adjusted to OD_{600}=0.2 and cells were grown at 30 ºC or 37 ºC for the indicated time. Serial dilutions (1/3) were plated on YPD and the resulting plates were photographed after 48 h of incubation at 30 ºC. To assess cell viability on plates, cells were grown in liquid cultures until mid-log phase. Equal cell numbers (2000 cells) were plated on YPD Agar plates supplemented or not with casamino acids (Vitamin Assay Casamino Acids, Difco) and incubated first at 38 ºC for 23 h (non permissive temperature) and then for 25 h at 30 ºC or incubated for 48 h at 30 ºC, as indicated in the figure panel. Note that the casamino acid mixture contains all amino acids except asparagine, cysteine, glutamine and tryptophan. Ura3-3 plasmid and assessment of protein degradation rates were as described previously (Lewis and Pelham, 2009). Protein extraction was as described in (von der Haar, 2007), with the addition of 10 mM *N*-ethylmaleimide (Sigma Aldrich) to inhibit deubiquitinating enzymes.

Proteasome activity

26S proteasome activity was assessed using 0.25 µg of rabbit proteasome (PW9310, Enzo Life Science) in 26S proteasome assay buffer (50 mM Tris□HCl, pH 7.5, 40 mM KCl, 5mM $MgCl_2$, 0.5 mM ATP, 1 mM DTT) and 100 µm of Suc-LLVY-amc, as described (Kisselev and Goldberg, 2005).

Supplemental References

Beraud, C., Sun, S. C., Ganchi, P., Ballard, D. W., and Greene, W. C. (1994). Human T-cell leukemia virus type I Tax associates with and is negatively regulated by the NF-kappa B2 p100 gene product: implications for viral latency. Mol Cell Biol *14*, 1374-1382.

Ghislain, M., Udvardy, A., and Mann, C. (1993). S. cerevisiae 26S protease mutants arrest cell division in G2/metaphase. Nature *366*, 358-362.

Harding, H. P., Novoa, I., Zhang, Y., Zeng, H., Schapira, M., and Ron, D. (2000). Regulated translation initiation controls stress-induced gene expression in mammalian cells. Mol Cell *6*, 1099-1108.

Kisselev, A. F., and Goldberg, A. L. (2005). Monitoring activity and inhibition of 26S proteasomes with fluorogenic peptide substrates. Methods Enzymol *398*, 364-378.

Lewis, M. J., and Pelham, H. R. (2009). Inefficient quality control of thermosensitive proteins on the plasma membrane. PLoS One *4*, e5038.

Narita, M., Young, A. R., Arakawa, S., Samarajiwa, S. A., Nakashima, T., Yoshida, S., Hong, S., Berry, L. S., Reichelt, S., Ferreira, M., Tavare, S., Inoki, K., Shimizu, S., and Narita, M. (2011). Spatial coupling of mTOR and autophagy augments secretory phenotypes. Science *332*, 966-970.

Onodera, J., and Ohsumi, Y. (2005). Autophagy is required for maintenance of amino acid levels and protein synthesis under nitrogen starvation. J Biol Chem *280*, 31582-31586.

Rousseau, E., Kojima, R., Hoffner, G., Djian, P., and Bertolotti, A. (2009). Misfolding of proteins with a polyglutamine expansion is facilitated by proteasomal chaperones. J Biol Chem *284*, 1917-1929.

Sancak, Y., Peterson, T. R., Shaul, Y. D., Lindquist, R. A., Thoreen, C. C., Bar-Peled, L., and Sabatini, D. M. (2008). The Rag GTPases bind raptor and mediate amino acid signaling to mTORC1. Science *320*, 1496-1501.

Thurston, T. L., Ryzhakov, G., Bloor, S., von Muhlinen, N., and Randow, F. (2009). The TBK1 adaptor and autophagy receptor NDP52 restricts the proliferation of ubiquitin-coated bacteria. Nat Immunol *10*, 1215-1221.

Vabulas, R. M., and Hartl, F. U. (2005). Protein synthesis upon acute nutrient restriction relies on proteasome function. Science *310*, 1960-1963.

von der Haar, T. (2007). Optimized protein extraction for quantitative proteomics of yeasts. PLoS One *2*, e1078.
